# Weights and Measures

**Published:** 1869-11

**Authors:** 


WEIGHTS AND MEASURES.—The metrical standard of weights and measures, according to a recent report to the Emperor Napoleon III, from the Minister of Agriculture and Commerce, is gaining ground in every country, and has been officially adopted by Belgium, Holland, Italy, the Papal States, Spain, Portugal, Greece, Mexico, Brazil, Chili, New Granada, and the republics of South America. An English commission, too, has declared in favor of its introduction into Great Britain. The minister proposes that a commission should be appointed for the purpose of delivering to foreign countries, metrical standards which may serve to render the system general.

We shall be glad to see it adopted in this country, and especially in the drug trade and the writing of prescriptions. Exact science in all its branches has recognized the superiority everywhere.

